# Traffic Sign Detection System for Locating Road Intersections and Roundabouts: The Chilean Case

**DOI:** 10.3390/s17061207

**Published:** 2017-05-25

**Authors:** Gabriel Villalón-Sepúlveda, Miguel Torres-Torriti, Marco Flores-Calero

**Affiliations:** 1 Departamento de Ingeniería Eléctrica, Pontificia Universidad Católica de Chile, Vicuña Mackenna 4860, Casilla 306-22, Santiago, Chile; procesosestocasticosespe@gmail.com (G.V.-S.); mtorrest@ing.puc.cl (M.T.-T.); 2 Departamento de Eléctrica y Electrónica, Universidad de las Fuerzas Armadas-ESPE, Av. Gral. Rumiñahui s/n, PBX 171-5-231B Sangolquí, Pichincha, Ecuador; 3 Departamento de Sistemas Inteligentes, Tecnologías *I*&*H*, CP 050102, Latacunga, Cotopaxi, Ecuador

**Keywords:** statistical template, traffic signs, color, road intersection, roundabouts, accidents

## Abstract

This paper presents a traffic sign detection method for signs close to road intersections and roundabouts, such as stop and yield (give way) signs. The proposed method relies on statistical templates built using color information for both segmentation and classification. The segmentation method uses the RGB-normalized (ErEgEb) color space for ROIs (Regions of Interest) generation based on a chromaticity filter, where templates at 10 scales are applied to the entire image. Templates consider the mean and standard deviation of normalized color of the traffic signs to build thresholding intervals where the expected color should lie for a given sign. The classification stage employs the information of the statistical templates over YCbCr and ErEgEb color spaces, for which the background has been previously removed by using a probability function that models the probability that the pixel corresponds to a sign given its chromaticity values. This work includes an analysis of the detection rate as a function of the distance between the vehicle and the sign. Such information is useful to validate the robustness of the approach and is often not included in the existing literature. The detection rates, as a function of distance, are compared to those of the well-known Viola–Jones method. The results show that for distances less than 48 m, the proposed method achieves a detection rate of 87.5% and 95.4% for yield and stop signs, respectively. For distances less than 30 m, the detection rate is 100% for both signs. The Viola–Jones approach has detection rates below 20% for distances between 30 and 48 m, and barely improves in the 20–30 m range with detection rates of up to 60%. Thus, the proposed method provides a robust alternative for intersection detection that relies on statistical color-based templates instead of shape information. The experiments employed videos of traffic signs taken in several streets of Santiago, Chile, using a research platform implemented at the Robotics and Automation Laboratory of PUC to develop driver assistance systems.

## 1. Introduction

Traffic accidents are the primary cause of death for young people between 15 and 29 years old. Between 20 to 50 million people are injured each year, while 1.3 million died due to traffic accidents, of which 91% take place in low and medium income countries [1,2,3]. Latin America is a region with high rates of road traffic accidents [2,4], due to diverse reasons which include driver education and behavior, law enforcement, and lack of adequate road infrastructure. However, technology can also play an important role in driver assistance systems that contribute to the alertness of the driver and better driving behaviors [5,6].

Most traffic accidents occur in urban areas, especially at road intersections and roundabouts. Country statistics for traffic accidents show that a significant number happened at road intersections, for instance, 22% in the USA [7,8], 58.7% in Japan during 1995 [9], 13.75% in Ecuador during 2015 [10], and 9.22% in Chile during 2014 [11]. Thus, the importance of developing systems for road intersections detection [12], which unlike other aspects such as pedestrian detection, lane-tracking and driver drowsiness or distraction [5,6] has not received enough attention. Prior work has focused on pavement segmentation to detect intersections [6] by analyzing the continuity and curvature of the road boundaries. However, occlusions in urban environments make the analysis of edges difficult or impossible. Therefore, the implementation of an advanced driving assistance system (ADAS) [13] requires a module capable of detecting road signs in general, and specifically those found at intersections.

We propose a traffic sign detection approach based on statistical templates built using normalized color information. The novelty of the approach lies in the probabilistic model of the sign (or object) conditioned over the intensity of the normalized color channels instead of using traditional shape descriptors. The results show that this approach is robust to variations in distance between the car and the traffic sign, as well as variation in illumination. Unlike deep learning techniques, the results show that it is possible to implement the proposed traffic sign detection approach with small datasets of a few hundred images.

This paper is organized as follows. First, the state-of-the-art of traffic sign detection algorithms is discussed in Section 2. Section 3 describes a new system for traffic sign detection and its modules based on color information. The experimental results, where an analysis between the detection rate and the distance is performed to verify the quality of this system, are presented in Section 4. Finally, Section 5 is devoted to the conclusions and discussion of future work.

## 2. State-of-the-Art

Traffic sign detection using visible-spectrum cameras may take different approaches. Some works implement feature classifiers. This means that a sliding-window method is used to compute features on different overlapping regions, which are then fed to the previously trained classifier [14,15]. The drawback of this strategy is that many positions and scales have to be tested using classifiers that may need computationally demanding training phases. More recent methods formulate a two-stage strategy, in which candidate or proposal regions are computed first by some “class-agnostic” segmentation process, i.e., extracting groups of pixels that share some characteristic without necessarily identifying whether they truly belong to the same class of object. In a second stage, some classification or decision process is used to complete the detection deciding whether some classes of objects sought are present or not. The methods proposed in [16,17,18,19,20,21,22,23,24] can be found among recent approaches for traffic sign detection employing regional proposal strategies together with classifiers. The most recent approaches to segmentation and classification employed in traffic sign detection are discussed next.

### 2.1. Segmentation for ROI Generation

In the context of traffic sign detection, blob generation and color analysis are the main techniques employed to segment regions of interest. Special efforts have been placed on making the color-based segmentation robust to large variations in illumination and weather conditions. Greenhalgh et al. [16] transform RGB into grayscale images using the red and the blue components and experimentally obtained thresholds to generate ROIs. Salti et al. [17] employ three color spaces derived from the RGB, the first to highlight road signs with a predominance of blue and red colors, the second one is for signs with intense red and the third one for the bright blue. Li et al. [18] have built the Gaussian space ($EE_{\lambda }E_{\lambda \lambda }$), where objects dominated by the green-red and blue-yellow colors are highlighted. The preselected regions are in turn transformed to normalized values $C_{\lambda }=E_{\lambda }/E$ and $C_{\lambda \lambda }=E_{\lambda \lambda }/E$, which are fed to a *k*-means clustering [25] to generate the ROIs. Zaklouta et al. [19] implement two RGB-based chromatic filters for ROIS generation, one for signs that have a red color prevalence, and another filter for red-yellow predominance; in both cases, thresholds are defined in terms of mean and variance. Lillo et al. [26] have used the L${}^{*}$a${}^{*}$b${}^{*}$ space to detect signs in which the blue, green, yellow and red colors dominate. Based on the *k*-means clustering algorithm, the authors build a classifier that employs the $a^{*}$ and $b^{*}$ components. Fleyeh et al. [23] use the *H* and *S* components of the HSV space to train a classifier and implement the color segmentation that yields ROIs. More recently, Han et al. [24] have used the *H* component of the HSI space, in which the traffic signs are highlighted in order to build a grayscale image where a set of ROIs is generated. Keser et al. [27] have used the HSV filter intervals to generate a set of ROIs. Finally, Zhu et al. [28] employ three different object proposal strategies (Selective Search, Edge Boxes and BING) and convolutional neural networks for classification, achieving an accuracy of 88% on average.

### 2.2. Recognition

The recognition stage typically employs feature classifiers, and therefore requires a feature descriptor and a classification algorithm. One of the most popular feature descriptors is the histogram of oriented gradients (HOG) [15], which provides information about objects’ shape. Recent works in traffic sign detection that employ the HOG descriptor include [16,17,19,29]. Li et al. [18] employ the PHOG descriptor, a variant of the HOG descriptor. Other descriptors are based upon the discrete Fourier transform [26], the Hough transform [23], the SURF method [30], the values of the neighboring pixels in a ROI [31], or predefined contour descriptors for basic shapes (circular, triangular, or rectangular) [27].

Concerning classifiers, most of the recent work in traffic sign detection employs SVM (support vector machine) classifiers [25]; see for example [16,17,18,19,26]. Another popular classification approach relies on artificial neural networks (NN). For example, recent work by Huang et al. [29] combines an NN-classifier with ELM (Extreme Learning Machine), and Pérez et al. [32] relies on MLPs (MultiLayer Perceptrons). The simpler *k*-NN (*k*-nearest neighbors) algorithm [25] is employed in the traffic sign detection method proposed in [24]. Recently, Deep Learning techniques are being used for simultaneous detection and recognition of traffic signs. CNN (Convolutional Neural Networks) is also employed in many of the most recent papers—Lau et al. [31], Jung et al. [33], Zeng et al. [34], Zhu et al. [28]—which propose new architectures for automatic sign detection. Other strategies, such as the one employed by Li and Yang [35] rely on a combination of DBM (Deep Boltzmann Machine) and CCA (Canonical Correlation Analysis) for feature extraction and classification. Lau et al. [31] have also experimented with RBNN (Radial Basis Neural Networks) for classification.

### 2.3. Databases

The main traffic sign databases correspond to the following countries: Germany [17,19,20,29,32], United Kingdom [16], Spain [22,26], Japan [36], China [28] or Malaysia [31]. Each country has its own regulations and standards concerning traffic signs, divided in regulatory, prevention and information categories [17,23,26,28,36]. Generally, they do not follow the Vienna Convention-Complaint for traffic signs [37].

Thus country-specific databases are required for the development of traffic sign detection systems. However, there is a lack of databases with traffic signs in Latin America. Therefore, another goal of this work is to contribute to the development of traffic sign detection systems that can be validated also on traffic signs of the Latin American region.

## 3. Proposed Approach for Segmentation and Recognition of Traffic Signs at Road Intersections and Roundabouts

The proposed computational strategy for detecting signs found at road intersections and roundabouts is composed of two parts. The first part generates ROIs in which traffic signs could be found calculating and analyzing color statistics in the normalized RGB space. The second part solves the recognition of signs in ROIs using a statistical template matching strategy using templates also in the normalized RGB space.

**Figure 1:**
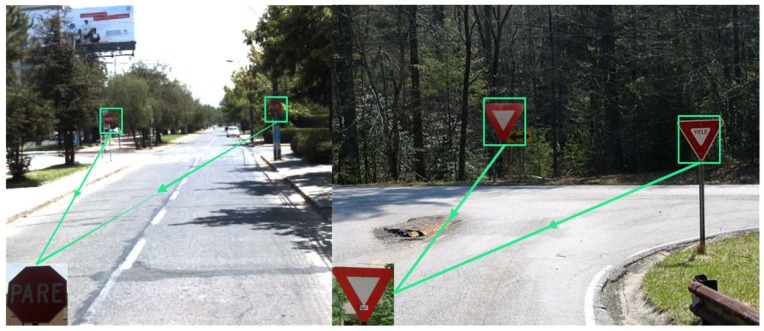
Examples of testing scenarios showing stop (**Left**) and yield (**Right**) signs near a road intersection and a roundabout.

The detection must be done at the furthest possible distance, so that the driver has enough time to react and to stop in time. Examples of typical testing scenarios for the proposed approach are presented in Figure 1, which shows a distant stop sign and a yield (give way) sign.

### 3.1. Chromaticity Filter for the Selection of ROIs

**Figure 2:**
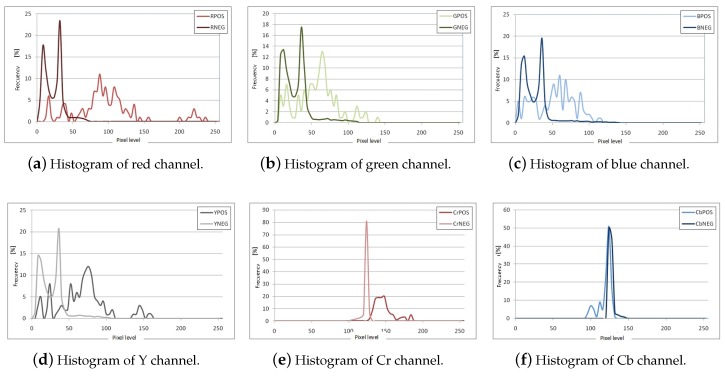

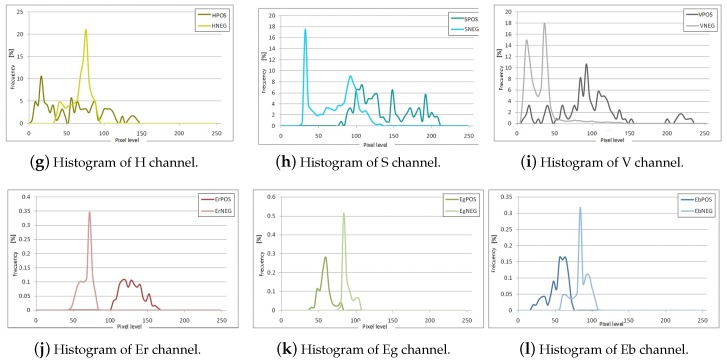
Histograms of the average Stop sign images (*c*POS) and their background (*c*NEG) for each channel *c* in the RGB, YCrCb, HSV and ErEgEb color spaces.

Under adequate illumination conditions, such as daylight or artificial lighting, the color of traffic signs is a feature that can be used to generate ROIs. Comparing the histograms of traffic signs in four color spaces, RGB, HSV, YCbCr and ErEgEb (the normalized RGB space) [38,39], it possible to observe in Figure 2 that some of the color spaces provide better discrimination capability between traffic signs and the image backgrounds. In Figure 2, the histograms labeled RPOS correspond to histograms of the stop sign, while the curves labeled RNEG correspond to the histograms of backgrounds or scenes that do not contain traffic signs.

The color space that shows the smaller overlap between the histograms of positives (signs) and negatives (non-sign) is the ErEgEb space, where in particular the Er channel shows a clear separation between the two classes. The Eg channel histograms have a small intersection, but likewise it serves to discard a significant portion of negatives. Finally, the Eb does not provide considerable information, but will be considered a part of the classification strategy, see Figure 2j–l. A similar analysis was conducted for the yield sign and the results allow to conclude that the ErEgEb space, and in particular Er and Eg channels provide a better discrimination capability.

**Figure 3:**
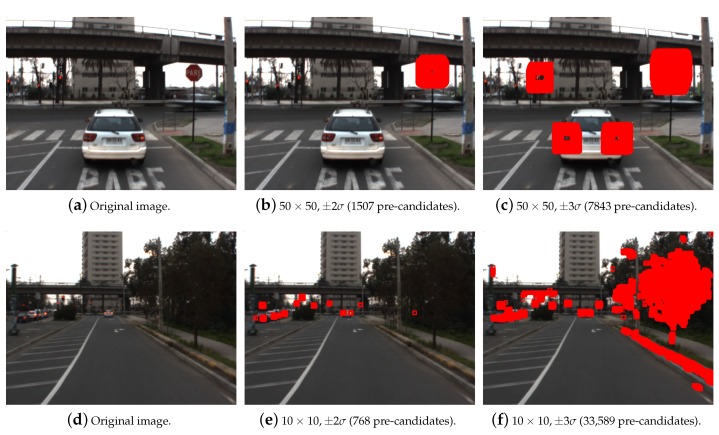
Examples of ROI (regions of interest) candidates generated for the stop sign using the chromaticity filter before region merging.

Therefore, the candidate regions of interest can be detected using a chromaticity filter, i.e., a filter that works on the variables that define color hue (dominant wavelength) and color purity or saturation (difference between the intensity of the dominant wavelength with respect to white, grey or black) regardless of luminance (magnitude of the color components vector) or psychological perception of illumination brightness or intensity (as an average of the components of the color vector). In other words, a chromaticity filter only requires two variables that describe dominant wavelengths regardless of the total energy by mapping the components of the thrichromacy color model into a subspace of two normalized values. Assuming a normal distribution of the chromaticity channels Er and Eg, the selection thresholds for extracting ROIs can be defined as intervals $\left[\mu_{c}-\alpha \sigma_{c},\mu_{c}+\alpha \sigma_{c}\right]$, c=Er,Eg, where $\mu_{c}$ and $\sigma_{c}$ are the mean and standard deviation of the channel *c* computed over a set of positives (images with traffic signs) according to:

(1a) $$\mu_{i,c}=\frac{\sum_{p}I_{i,c}\left[p\right]}{N_{P}},$$

(1b) $$\mu_{c}=\frac{\sum_{i}\mu_{i,c}}{N_{I}},$$

(1c) $$\sigma_{c}^{2}=\frac{\sum_{i}{\left(\mu_{i,c}-\mu_{c}\right)}^{2}}{N_{I}}.$$

where $I_{i,c}\left[p\right]$ is the value of channel *c* at the pixel location *p* within the traffic sign of the *i*-th reference image (positive), $i=1,2,\ldots ,N_{I}$, $N_{I}$ is the number of positive images, $N_{P}$ is the number of pixels within the reference traffic sign area, $\mu_{i,c}$ is the mean value of channel *c* for the *i*-th image, $\mu_{c}$ is mean value of channel *c* across the ensemble of $N_{I}$ images, and $\mu_{c}^{2}$ is the variance of the mean values $\mu_{i,c}$, $i=1,2,\ldots ,N_{I}$. The value α is set to minimize false positives and false negatives that will be passed to the recognition stage. A practical value that ensures the lowest amount of false positives while preventing misdetections was found to be α=2. It is to be noted that using the so-called summed integral area tables or integral images [14,40] is possible to reduce the computation time of the average values on N×N sliding blocks. Two examples showing the generation of preliminary candidates for ROIs using windows sizes of 50×50 and 10×10 pixels are shown in Figure 3 for values of α=2,3.

**Figure 4:**
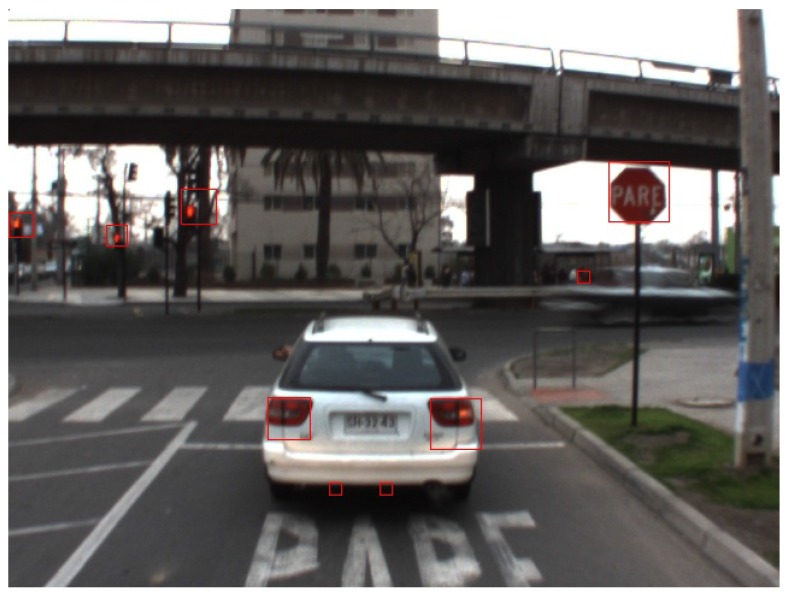
Example of ROIs obtained after removal/merging of overlapping pre-candidates selected with the chromaticity filter.

The last step for the final proposal of ROIs is to eliminate the overpopulation of candidates. To this end, all the candidates that are contained within or are a sub-window of another candidate are discarded, so that only the largest block remains. Next, windows whose mean value is closest to $\mu_{c}$ in each neighborhood are selected so that there is only one candidate per neighborhood. Figure 4 shows the final ROI proposal obtained using 10 window sizes ranging from 10×10 to 50×50 in geometric progression, with a fixed scaling factor between each size. The number of preliminary candidates satisfying the chromaticity filter threshold was 32,849. This number is reduced to only 9 after the pre-candidate selection and merging step.

### 3.2. Recognition of Traffic Signs Based on Statistical Templates

**Figure 5:**
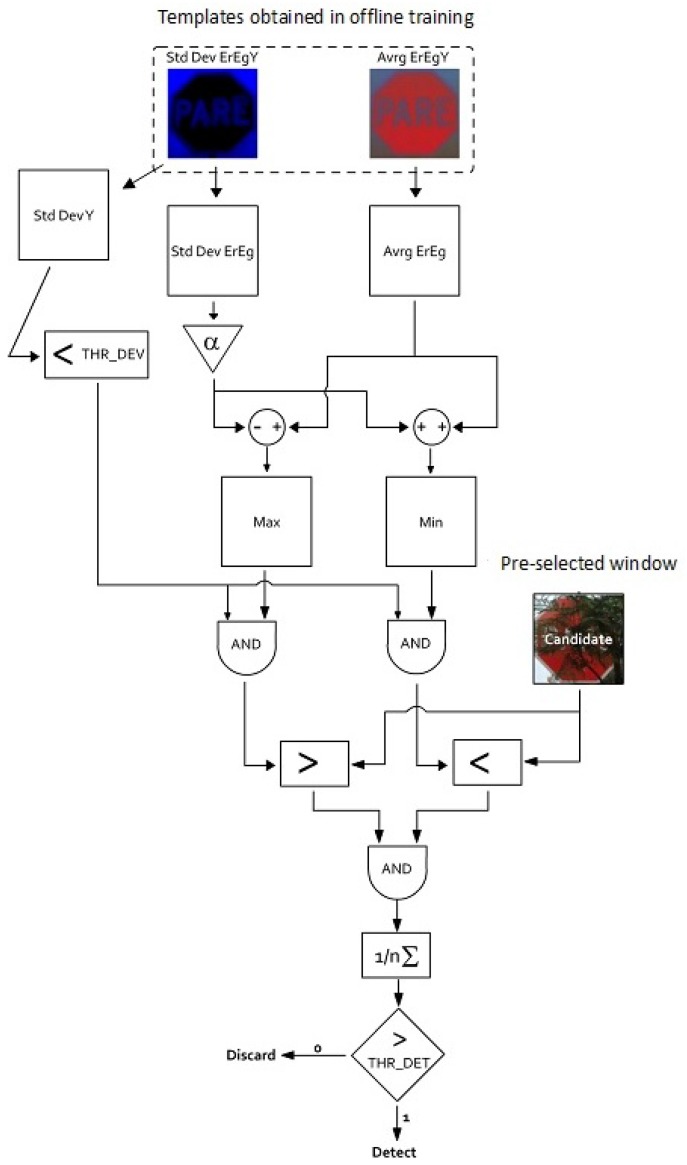
Flow chart of the recognition stage based on statistical templates.

The second stage of the proposed traffic sign detection approach is responsible for solving the identification of ROIs as traffic signs of a given type. To this end, a set of images is employed to create two statistical models, one with the mean intensity and the other with its standard deviation for each pixel belonging to the traffic sign. Testing on a sliding block for the percentage of pixels that fall within the expected intensity range for a given location provides a discriminator to detect traffic signs from background and non-traffic sign objects. A flow diagram of the proposed method is shown in Figure 5. The corresponding pseudocode of the algorithm is presented in Algorithm 1. The most effective recognition of traffic signs is achieved applying the algorithm to the Er and Eb channels of the ErEgEb color space. Only two channels conveying the chromaticity information are sufficient because the magnitude normalization satisfies Er+Eg+Eb=1, making the third channel dependent on the other two (Eg=1−Er−Eb). The probabilistic model that defines the discrimination thresholds is discussed next.

In order to develop the probabilistic template matching model to recognize traffic signs, it is first convenient to introduce the following notation:

- $I≜\left\{I[k],k=1,..,n\right\}$ is a block or subwindow composed of pixel values I[k],
- I[k] is a vector with the pixel chromaticity components Er and Eb, k=1,..,n,
- $O≜\left\{O[k],k=1,..,n\right\}$ is the object (sign) class label of subwindow *I*,
- O[k]: the object (sign) class label at every pixel *k*, k=1,..,n in the subwindow *I*.

The probability that a window corresponds to a particular object (sign) is:

(2) $$\begin{array}{cc} P\left(O|I\right) & =1-P\left(\bar{O}|I\left[1\right],I\left[2\right],...,I\left[n\right]\right),\\ & =1-\prod_{k=1}^{n}P(\bar{O}\left[k\right]|I\left[k\right]),\\ & =1-\prod_{k=1}^{n}\left[1-P\left(O[k]|I[k]\right)\right], \end{array}$$

where $\bar{O}$ is the complement of *O* and $P\left(\bar{O}\left[k\right]|I\left[1\right],..,I\left[n\right]\right)=P\left(\bar{O}\left[k\right]|I\left[k\right]\right)$ is obtained by assuming independence between $\bar{O}\left[k\right]$ and I[j], for all j≠k. This is possible in view of the fact that *O* and *I* are random samples [41,42]. Also, this means that the probability of a pixel not belonging to an object only depends on the pixel value and not its neighborhood. In other words, the background (non-sign) pixels are assumed to be conditionally independent with respect to their neighborhood. This assumption is not entirely true in every area of the background, but simplifies the probability computation.

**Algorithm 1:** Traffic sign recognition algorithm based on statistical templates**Input**: $I_{c}$: candidate image block, α: pixel acceptance amplitude parameter, $\lambda_{\sigma }$: background pixels discard threshold, $\lambda_{det}$: minimal amount of pixels threshold for detection. **Output**: $Z_{det}$: binary detection output. // Loading pre-trained masks ${\bar{A}}_{M}=$
LoadAverageMask(); $\sigma_{M}=$
LoadStandardDeviationMask(); // Pixel mask discarding corresponding to the background $B_{M}=\sigma_{M}Y<\lambda_{\sigma }$; // Minimum and maximum accepted masks $MAX={\bar{A}}_{M}+\alpha \times \sigma_{M}$; $MIN={\bar{A}}_{M}-\alpha \times \sigma_{M}$; // Pixel mask accepted $P_{M}=\left(MIN\leq I_{c}\leq MAX\right)\times B_{M}$; // Final decision **if**
SumPixels($P_{M}$)/SumPixels($B_{M}$) $\geq \lambda_{det}$
**then**  $Z_{det}=true$; **else**  $Z_{det}=false$; **end**

In order to compute the posterior probability $P\left(O[k]|I[k]\right)$ that a pixel *k* has label O[k] given its chromaticity value I[k], Bayes’ theorem is used

(3) $$P\left(O[k]|I[k]\right)=P\left(I[k]|O[k]\right)\frac{P\left(O[k]\right)}{P\left(I[k]\right)}$$

to express the posterior probability in terms of the measurement model $P\left(I[k]|O[k]\right)$.

The measurement model $P\left(I[k]|O[k]\right)$ can be obtained assuming that the pixel values of the object (sign) of interest follow a normal distribution $N(\mu_{Ex},\sigma_{Ex})$, x=r,b, with mean chromaticity $\mu_{Ex}$ and standard deviation $\sigma_{Ex}$ obtained from a set of reference images, see the last row of Figure 2.

The likelihood or conditional probability that the measured chromaticity values (Er[k],Eg[k]) at pixel *k* take some value in the interval $\left[Ex\left[k\right]-\beta \sigma_{Ex}\left[k\right],Ex\left[k\right]+\beta \sigma_{Ex}\left[k\right]\right]$, x=r,g, given object class, is then given by:

(4) $$\begin{array}{ccc} P(I[k]=(Er[k],Eb[k])|O[k]) & = & \prod_{x=r,b}\frac{2}{\sqrt{\pi }}\int_{0}^{\frac{E_{x}\left[k\right]+\beta \sigma_{Ex}\left[k\right]-\mu_{Ex}\left[k\right]}{\sigma_{Ex}\left[k\right]\sqrt{2}}}e^{-t^{2}}dt\\ & = & \prod_{x=r,b}\mathrm{erf}\left(\frac{E_{x}\left[k\right]+\beta \sigma_{Ex}\left[k\right]-\mu_{Ex}\left[k\right]}{\sigma_{Ex}\left[k\right]\sqrt{2}}\right) \end{array}$$

The prior probability $P\left(O[k]\right)$ of finding the object (sign) in an image can be obtained experimentally from the set of reference images as:

(5) $$P\left(O[k]\right)=\frac{TP}{TP+FP},$$

where TP is the true positive rate and FP is the false positive rate for the object (sign) of class (type) *O*. This ratio is known as a positive predictive value and it describes the probability of traffic signs being correctly detected [43].

Finally, the probability of the occurrence of chromaticity values P(I[k]=(Er,Eb)) can be obtained empirically or analytically. The empirical approach would require constructing the histograms for the Er and Eb channels using a representative set of training data and normalizing the histograms to obtain the ratio between the number of pixels with the given chromaticity levels with respect to the the total amount of pixels. Analytically, a cumulative density function for the values of each channel can be deduced under the assumption that RGB values distribute according to a uniform distribution within a window block that contains both object and background pixels (see appendix for calculations details). The cumulative function for the chromaticity values under the uniform distribution assumption is given by:

(6) $$F\left(y\right)=\left\{\begin{array}{cc} 0 & y\leq 0\\ \frac{y}{1-y} & 0<y\leq \frac{1}{3}\\ \frac{-21y^{3}+27y^{2}-9y+1}{6y^{2}\left(1-y\right)} & \frac{1}{3}<y\leq \frac{1}{2}\\ \frac{5y^{2}+2y-1}{6y^{2}} & \frac{1}{2}<y<1\\ 1 & y\geq 1 \end{array}\right.,$$

**Figure 6:**
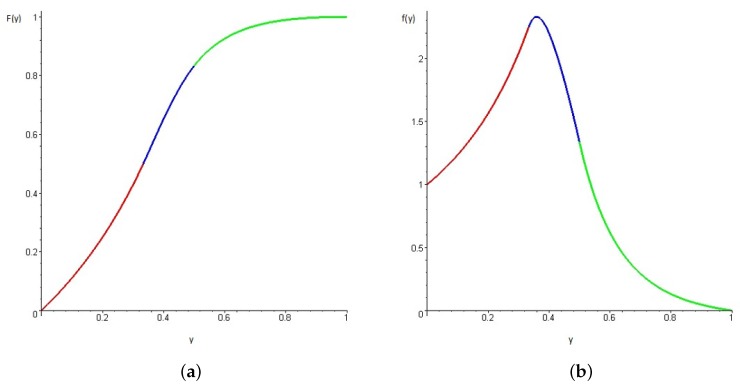
Background probability model, cumulative density function F(y) (**a**), and probability density function f(y) (**b**), for ErEgEb space, considering that each channel of RGB space follows a uniform distribution.

The probability density function f(y) associated to the cumulative distribution of the chromaticity values I[k]=(Er,Eb) is easily obtained from (6) by deriving F(y) with respect to *y*. Figure 6 depicts the cumulative and density functions. The density function f(y) reaches a maximum at y∼0.36, which is the most likely background value without prior knowledge of the object (sign) class. Thus, it is desirable that the objects of interest have their chromaticity levels far away from this value.

The analysis thus far provides enough tools to compute the probability that a window corresponds to the object of interest using (2) and the statistical templates for the mean and standard deviation $\mu_{Ex}\left[k\right],\sigma_{Ex}\left[k\right]$ over the window block k=1,2,…,n. However, these templates consider both the pixels of the object of interest and the background; therefore, to improve discrimination, it is convenient to discard background pixels.

**Figure 7:**
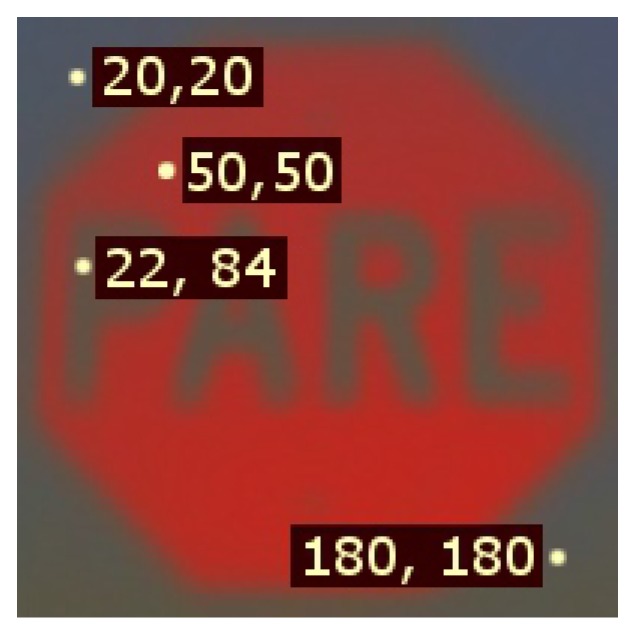
Representative points of a stop sign template obtained by averaging reference samples in the ErEgEb space.

**Figure 8:**
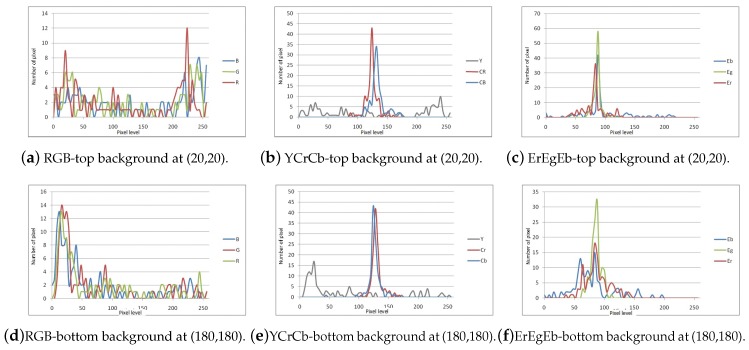

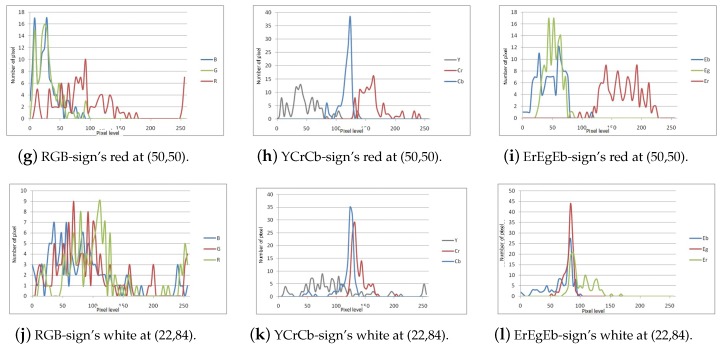
Histograms in three color spaces for pixels in the reference areas of Figure 7.

**Figure 9:**
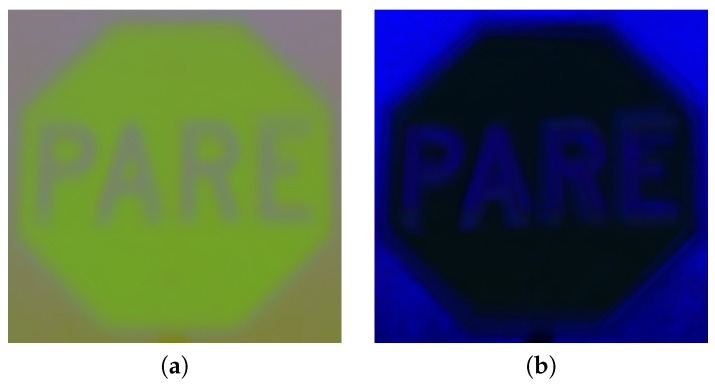
Scaled images of the mean $\mu_{Y}$ (**a**) and standard deviation $\sigma_{Y}$ (**b**) of the Y channel.

To this end, Figure 7 presents the representative points to the comparison of histograms of the pixels corresponding to the background and to the object of interest presented in Figure 8 reveals that the luminance channel Y spreads over the entire range of possible values for background objects. Hence, the standard deviation of the luminance channel is a good indicator to determine whether the pixel is part of the object of interest or part of the background. Figure 9 shows the mean and standard deviation for the luminance channel Y. It is clear that the template built using the variance provides higher contrast between background and foreground than using the mean value of the luminance to create a mask for discarding background regions.

**Figure 10:**
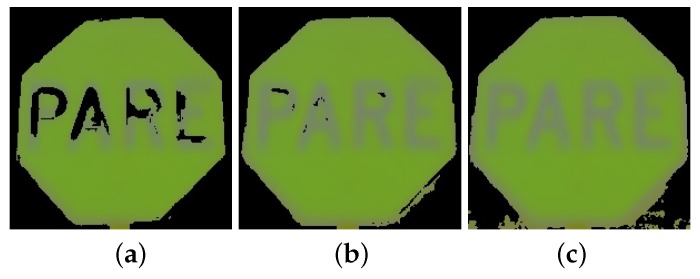
Discarding the background in the Y channel with different thresholds: (**a**) $\sigma_{Y}=55$; (**b**) $\sigma_{Y}\;=\;60$; and (**c**) $\sigma_{Y}=65$.

To create a mask for discarding background pixels, an adequate thresholding value for the standard deviation template is $\sigma_{Y}=60$ as may be observed in Figure 10 since it allows to retain most of the pixels of the object of interest and discard all of the background. Assuming the luminance Y would distribute Gaussianly, $\sigma_{Y}>60$ would imply that 95% of the samples would fall within ±120 intensity levels, thus it would cover a range of 240 levels, which would be almost the full range for an 8-bit image with 255 levels. Thus, higher values for the threshold on $\sigma_{Y}$ are not convenient, while lower values cause part of the object to be labeled as background, as shown in Figure 10a with $\sigma_{Y}=55$.

By the foregoing, the channels to be used in the recognition stage have the luminance to remove the background and the chromaticity values Er and Eb to confirm the ROIs are of a given traffic sign, ensuring robustness to illumination variations.

## 4. Testing Methodology and Experimental Results

### 4.1. Perception and Processing Systems

**Figure 11:**
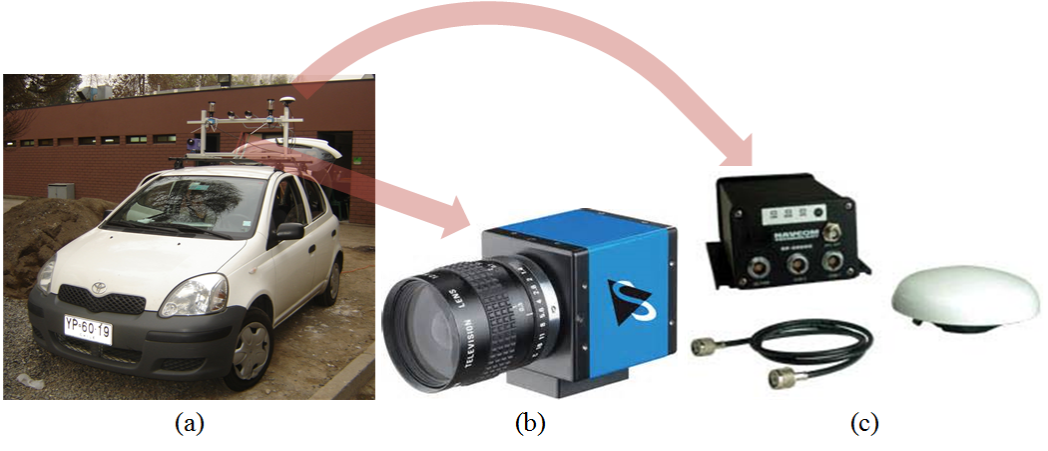
Experimental platform, vehicle (**a**); camera (**b**); and GPS (**c**).

The vehicle shown in Figure 11a was employed as a testing platform for the experiments. The system comprises several cameras (visible spectrum, IR, catadioptric), an IMU and a RTK GPS; see [6] for further details. For the experiments presented here, only one camera from Imaging Source, model DFK31BF03 model (see Figure 11b) was used together with the RTK GPS from Navcom Technology, model SF-2050, with decimeter positioning accuracy (see Figure 11c). The camera has a resolution of 1024 × 768 pixels and delivers images at 30 fps, while the GPS sampling rate is 10 Hz. Thus, the GPS data was interpolated to match the sampling instants of the camera. Registering the position is important in order to compute detection rates as a distance function. The processing of the images was carried out on a PC with an Intel Core 2 Duo processor with a 2.0 GHz frequency, and 3.5 GB of RAM. All the algorithms were implemented in C++ using the OpenCV version 2.2 library.

### 4.2. Training and Validation Dataset

**Figure 12:**
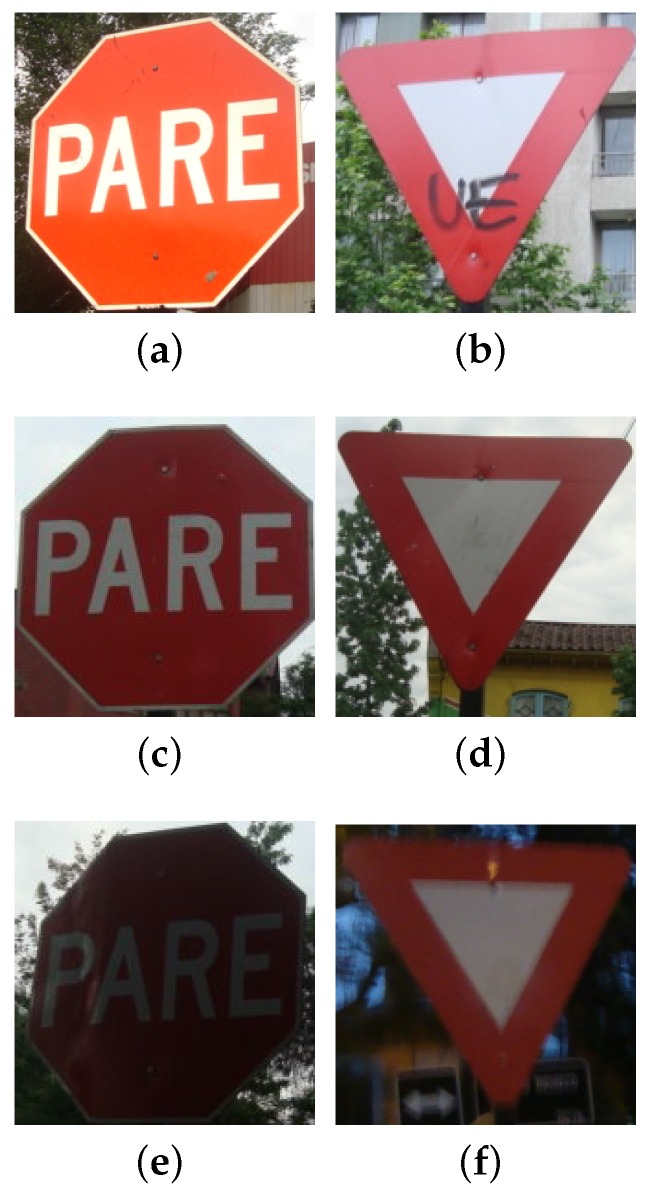
Typical traffic signs of road intersections and roundabouts in Chile: stop and yield signs; under different lighting conditions (sunny (**a**,**b**), normal (**c**,**d**) and dark (**e**,**f**)) and observer positions.

The training dataset contains 2567 negative examples, 122 images containing stops signs and 80 images containing yield signs. The positive samples were randomly rotated, scaled and translated in order to produce 7000 positive examples; see Figure 12. The validation dataset contains 273 stop signs and 447 yield signs captured in six different driving runs.

The datasets consider a sequence of signs as the car approaches different intersections in the city of Santiago, Chile. Both datasets contain traffic signs in real driving conditions, under varying illumination and partial occlusion.

The database has been made available at RAL [44] to contribute to the making of new studies and the development of ADAS.

### 4.3. Experiments Employing the Viola–Jones Method and the Proposed Statistical Template Approach

In this section, the proposed traffic sign detection approach based on statistical templates is tested and compared to the well-know Viola–Jones method [45].

#### 4.3.1. Viola–Jones Method:

The detection rates of the Viola–Jones approach for the stop and yield signs are summarized in Table 1 as a function of distance. The detection rate for stop signs is 100% when distances are 20 m or less. However, the detection rate rapidly falls to 0% for distances above 35 m. On the other hand, less that 3.1% of the yield signs was detected for distances below 20 m. For distances above 20 m, it was not possible to detect any yield sign. This is attributed, in part, to the fact that some samples in the dataset contained signs with marks and graffiti on it. These results show that the Viola–Jones approach is highly sensitive to possible modifications in the signs sought. The false alarm rates of the Viola–Jones approach for the stop and yield signs were practically 0%, as shown in Table 2.

#### 4.3.2. Statistical Template Method:

**Figure 13:**
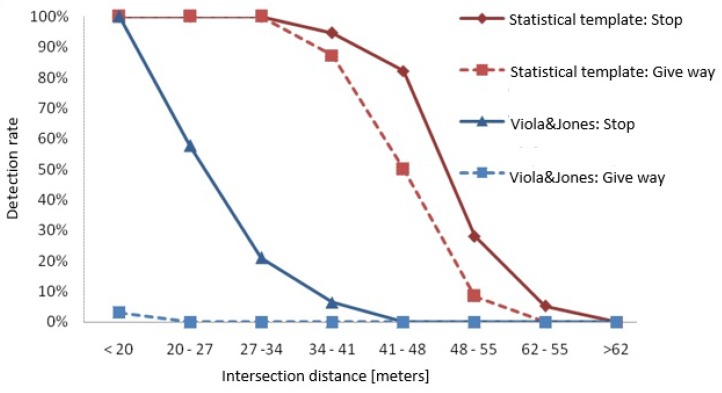
Comparison of detection rates versus distance between the Viola–Jones method and the statistical templates method for the stop and yield signs.

The proposed algorithm was executed employing eight different templates sized from 14×14 to 50×50 pixels in geometric progression. The detection rates presented in Table 3 show that a high rate of success was achieved for distances up to 37 m in the case of the stop sign and 35 in the case of the yield sign. Detection rates fall to 0% for distances above 52 m in the case of the stop sign and 48 m in the case of the yield sign. Unlike the Viola–Jones approach, false alarm rates were between 3.6% and 6.9% as shown in Table 2. A comparison of the detection rate performance of the proposed method with the Viola–Jones approach is presented in Figure 13. This figure shows the effectiveness of the proposed approach at detecting traffic signs earlier than the Viola–Jones method does.

Table 1 and Table 3 were constructed with approximately 430 images for each distance class.

In terms of the computational effort, the Viola–Jones method required 450 ms per frame while the proposed approach required 950 ms per frame. This amount could be decreased for real-time operation using dedicated graphic processing units (GPUs).

**Figure 14:**
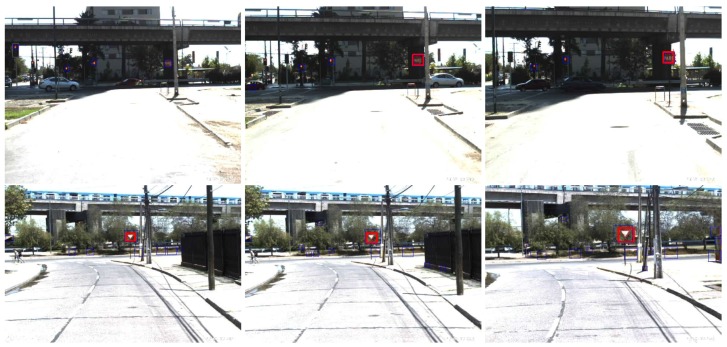
Example of the proposed system in different instants of time, in daytime conditions. Stop sign (**Top**) and Yield sign (**Bottom**), where blue indicates the ROIs and red shows the true sign.

Finally, Figure 14 presents an extended example of the proposed system, in real driving conditions during the day, for both stop and yield signs.

## 5. Conclusions

This paper presented an approach based on statistical templates computed from chromaticity and luminance values for traffic sign detection near road intersections and roundabouts. The approach is evaluated using a dataset of stop and yield (give way) signs from Chile and compared to the well-known Viola–Jones classification method.

The proposed approach is divided into two stages. The first stage is a segmentation stage based on the chromaticity filter applied to the Er and Eg channels of the ErEgEb color space (the normalized RGB color space). In the second stage, the chromaticity filter returns candidate regions that are responsible for the recognition of traffic signs and which must be tested. The selection of the Er and Eg channels is based on the analysis of the histograms of the color components in four color spaces (RGB, YCrCB, HSV and ErEgEb), which shows that the ErEgEb provides greater capacity to discriminate candidate traffic signs. The segmentation stage employs a selection threshold that can be computed automatically from a reference dataset. The recognition stage is based on a statistical template built with information of the Er and Eb chromaticity channels and the *Y* luminance channel. The luminance channel is employed to create a traffic sign mask from the variance of pixels that allows background regions within a ROI to be discarded. The Er and Eb channels are used to compute a statistical template that provides the sign selection thresholds. A probabilistic model and probability distribution function were derived to construct a recognition process based on Bayesian inference.

The results obtained show that the proposed approach has higher detection rates than the Viola–Jones method. The experiments considered the evaluation of the detection rate at different distances as the vehicle approaches a traffic sign. On average, the proposed approach exhibits a detection rate of 87.5% for yield signs and 95.4% for stop signs at distances below 48 m.

The two main advantages of the proposed approach are summarized in that it does not require a computationally expensive training or calibration stage, and it is not sensitive to changes in illumination, partial occlusion or marks drawn on the traffic sign. Future work will consider extending the proposed approach to the detection of traffic lights at road junctions and crosswalks, as well as to the detection of other signs not necessarily found at road intersections. Ongoing research is considering joint analysis of lane geometry analysis and edge continuity together with traffic sign detection to improve the detection of road intersections. The main limitation of this model is the computing time, which is about 950 ms per frame. As a future work, we will work to reduce this processing time.

## Figures and Tables

**Table 1:** Detection rate based on the Viola–Jones method.

Distance to the Intersection [mt]	Yield %	Stop %
>62	0.0%	0.0%
62–55	0.0%	0.0%
55–48	0.0%	0.0%
48–41	0.0%	0.0%
41–34	0.0%	6.5%
34–27	0.0%	21.0%
27–20	0.0%	57.6%
<20	3.1%	100.0%

**Table 2:** False alarm rate per frame.

Method	Yield	Stop
Viola–Jones	0.006	0.0
Statistical template	0.036	0.069

**Table 3:** Detection rate based on the statistical template method.

Distance to the Intersection [mt]	Yield %	Stop %
>62	0.0%	0.0%
62–55	0.0%	5.2%
55–48	8.5%	28.1%
48–41	50.0%	82.2%
41–34	87.3%	94.7%
34–27	100.0%	100.0%
27–20	100.0%	100.0%
<20	100.0%	100.0%

**Figure A1:**
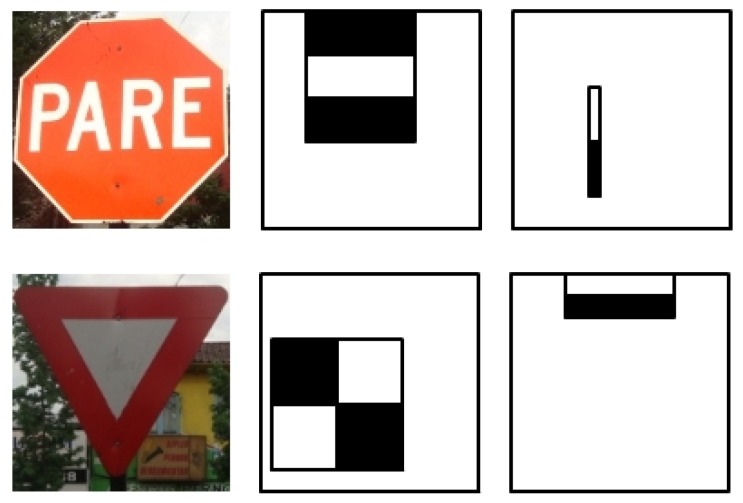
First and second Haar-like convolution masks for stop and yield signs employed in this work.

## Appendix A. Deduction of the Background Probability Distribution

To derive the cumulative density function of chromaticity values for any pixel F(y) in Equation (6), it is assumed that each channel of the RGB color spaces follows a uniform distribution. Thus, three random variables $X_{1}$, $X_{2}$ and $X_{3}$ representing each channel are defined according to the uniform distribution:

(A1) $$X_{1},X_{2},X_{3}∼U\left(0,S\right)\;\Rightarrow f\left(x\right)=\left\{\begin{array}{cc} 0 & x<0\vee x\geq S\\ \frac{1}{S} & 0\leq x<S \end{array}\right.,$$

The cumulative distribution function of the chromaticity channel $E_{X_{1}}=X_{1}/\left(X_{1}+X_{2}+X_{3}\right)$, given by:

(A2) $$F\left(y\right)=P\left(Y\leq y\right)=P\left(\frac{X_{1}}{X_{1}+X_{2}+X_{3}}\leq y\right),$$

is calculated as follows. The procedure for $E_{X_{2}}$ or $E_{X_{3}}$ is exactly the same.

In finding a closed-form expression for (A2), the following cases are considered:

- Case y=0:
  (A3) $$\begin{array}{ccc} F(0) & = & P\left(\frac{X_{1}}{X_{1}+X_{2}+X_{3}}\leq 0\right)\\ & = & P\left(X_{1}\leq 0\right)=0. \end{array}$$
- Case y=1:
  (A4) $$\begin{array}{ccc} F(1) & = & P\left(\frac{X_{1}}{X_{1}+X_{2}+X_{3}}\leq 1\right)\\ & = & P\left(X_{2}+X_{3}\geq 0\right))=1. \end{array}$$
- Case 0<y<1:
  (A5) $$\begin{array}{ccc} F(y) & = & P\left(\frac{X_{1}}{X_{1}+X_{2}+X_{3}}\leq y\right)\\ & = & P\left(X_{1}\leq \frac{y}{1-y}\left(X_{2}+X_{3}\right)\right). \end{array}$$

The last case amounts to solving the following integral:

(A6) $$F\left(y\right)=\int_{0}^{S}f\left(x_{3}\right)\int_{0}^{S}f\left(x_{2}\right)\int_{0}^{\frac{y}{1-y}\left(x_{2}+x_{3}\right)}f\left(x_{1}\right)dx_{1}dx_{2}dx_{3}.$$

The inner integral of $f(x_{1})$ must consider that if $\frac{y}{1-y}\left(x_{2}+x_{3}\right)\geq S$, then density function $f\left(x_{1}\right)=\frac{1}{S}$ is integrated up to *S*, while if $\frac{y}{1-y}\left(x_{2}+x_{3}\right)<S$, then the integral is computed for $x_{1}\in \left[0,\frac{y}{1-y}\left(x_{2}+x_{3}\right)\right]$. For the latter to be fulfilled throughout the non-zero domain of $f(x_{2})$ and $f(x_{3})$, the following must be satisfied:

(A7) $$\frac{y}{1-y}\left(x_{2}+x_{3}\right)\leq S\;\forall x_{2},x_{3}\in \left[0,S\right]\Rightarrow y\leq \frac{1}{3}$$

Similarly, when analyzing the limits of integration for the integrals over $x_{2}$ and $X_{3}$, so that the non-null parts are integrated and the result is continuous over the range of integration, the following intervals are obtained for *y*: $\left(0,\frac{1}{3}\right]$, $\left(\frac{1}{3},\frac{1}{2}\right]$ and $\left(\frac{1}{2},1\right)$.

For $0<y\leq \frac{1}{3}$:

(A8) $$F\left(y\right)=\int_{0}^{S}\frac{1}{S}\int_{0}^{S}\frac{1}{S}\int_{0}^{\frac{y}{1-y}\left(x_{2}+x_{3}\right)}\frac{1}{S}dx_{1}dx_{2}dx_{3}=\frac{y}{1-y},$$

For $\frac{1}{3}<y\leq \frac{1}{2}$, the integral is calculated as follows:

(A9) $$\begin{array}{cc} F(y) & =\int_{0}^{\frac{1-y}{y}S-S}\frac{1}{S}\int_{0}^{S}\frac{1}{S}\int_{0}^{\frac{y}{1-y}\left(x_{2}+x_{3}\right)}\frac{1}{S}dx_{1}dx_{2}dx_{3}\\ & +\int_{\frac{1-y}{y}S-S}^{S}\frac{1}{S}\int_{0}^{\frac{1-y}{y}S-x_{3}}\frac{1}{S}\int_{0}^{\frac{y}{1-y}\left(x_{2}+x_{3}\right)}\frac{1}{S}dx_{1}dx_{2}dx_{3}\\ & +\int_{\frac{1-y}{y}S-S}^{S}\frac{1}{S}\int_{\frac{1-y}{y}S-x_{3}}^{S}\frac{1}{S}\int_{0}^{S}\frac{1}{S}dx_{1}dx_{2}dx_{3}\\ & =\frac{-21y^{3}+27y^{2}-9y+1}{6y^{2}\left(1-y\right)}, \end{array}$$

Finally, for $\frac{1}{2}<y<1$, the integral is computed as follows:

(A10) $$\begin{array}{cc} F(y) & =\int_{0}^{\frac{1-y}{y}S}\frac{1}{S}\int_{0}^{\frac{1-y}{y}S-x_{3}}\frac{1}{S}\int_{0}^{\frac{y}{1-y}\left(x_{2}+x_{3}\right)}\frac{1}{S}dx_{1}dx_{2}dx_{3}\\ & +\int_{0}^{\frac{1-y}{y}S}\frac{1}{S}\int_{\frac{1-y}{y}S-x_{3}}^{S}\frac{1}{S}\int_{0}^{S}\frac{1}{S}dx_{1}dx_{2}dx_{3}\\ & +\int_{\frac{1-y}{y}S}^{S}\frac{1}{S}\int_{0}^{S}\frac{1}{S}\int_{0}^{S}\frac{1}{S}dx_{1}dx_{2}dx_{3}\\ & =\frac{5y^{2}+2y-1}{6y^{2}}. \end{array}$$

The above results in the cumulative distribution presented in Equation (6).

## Appendix B. Traffic Sign Detection by Using the Viola–Jones Method

The Viola–Jones object recognition approach [14,40] has been used in multiple computer vision applications [45,46,47]. In this work, it has been used to build a traffic signs detector, trained for stop and yield signs. Figure A1 shows the first and second convolution masks based on Haar-like features for each of the traffic signs considered in this work.
